# Annotations

**Published:** 1888-07-28

**Authors:** 


### ANNOTATIONS.

**The Extension of the Leeds Infirmary**

THE inhabitants of Leeds purpose enlarging their infirmary. When north-country folks purpose a thing, they mean something more than that they would like it done, if somehow, without trouble to themselves, it would get itself accomplished; they mean that they will do it thoroughly, and do it themselves. There is not in the country a town where there is a more general interest taken in the local hospital than Leeds. We have several times had to chronicle in our columns the munificent gifts of the working classes to the Infirmary, and the steadily ascending sum of workmen's yearly contributions; but the rich inhabitants of Leeds are worthy of their poorer fellow-citizens, and we pay them no small compliment in saying so. At present the Leeds Infirmary contains 320 beds, and the "Ida" Semi-Convalescent Hospital, recently opened at Cookridge in connection with it, holds 42; but this is hardly sufficient for the needs of a population so large a proportion of whom look, and look with reason, to the Infirmary as their proper home in sickness, and who are liable to those accidents which, in spite of all precautions, are apt to occur in factories. Therefore the Board of Management contemplate an extension of the institution, calculated to cost £30,000, "which" says Mr. Thomas Blair (the general manager of the Infirmary), "I believe we shall get in a short time." He does not speak so hopefully without good reason. At the last meeting of the Weekly Board of the Infirmary the chairman announced the receipt of the following contributions to the extension fund, in addition to a gift, previously announced, of £5,000 from Colonel North: Sir James Kitson, £2,500; Mr. W. Beckett, M.P., £1,000; Messrs. William Williams Brown, and Co., £1,000; Mr. A. Pickard, £1,000; Messrs. Joshua Tetley and Sons, £1,000; Mr. R. B. Jowitt, £500; Mr. E. Schunck, £500; Anonymous, £500; Mr. James Stables, £525; Mrs. Stables, £525; Miss Stables, £525; and Miss Lucy Stables, £525—making a total, with Colonel North's subscription, of £15,100. No wonder Mr. Blair speaks hopefully! And such donations as these have a double effect. Not only do they facilitate the good work the Board of Management have undertaken, but they encourage the poorer inhabitants of Leeds to persevere in the generosity they have hitherto shown. A workman will give his pence more readily when he sees that his master gives proportionately out of his abundance; and thus rich and poor encourage each other in a noble emulation in all good works.

**Roses in the Rain.**

A DAY of sharp thunder-showers, with dazzling spells of sunshine between, was the day, when the Princess Louise opened the flower show at the National Hospital for the Paralysed and Epileptic. One could hardly predict what the next half-hour would be like, and while one was hoping for a little brightness a new storm came on. Not royal weather exactly, but not wholly inappropriate for a fête at an institution where so many of the inmates fluctuate between apparently perfect health and a condition with which the sharpest atmospheric storms may fitly compare. But whatever weather there was outside the hospital, there was brightness within—flowers, and music, and gracious words. Not a patient in the hospital but felt that it would be a double misfortune if any ailment of his disturbed the success of that festival day; not a nurse or official but was determined to show the Princess, and everyone else, that there was a bright side to this palace of pain. And surely there is! Pain is one of the deepest things in our nature, and is the mother of many of the noblest—patience, gratitude, and faith on the one hand, and tenderness and helpful loving care and help on the other—things that bring both nurse and patient nearer to the Christ who went about doing good, yet when His time came did not evade His human inheritance of sorrow and pain. There are not many who have suffered in soul or body who will not say, even on this earth, "It was good for me that I have been afflicted"; elsewhere, in the world where all things shall be made clear, we may see that only through pain could the peace and gladness of Heaven be won. Meanwhile we know that though the storms of life beat harshly upon us, love and pity rear for us peaceful shelters, where the roses of charity and tenderness bloom fragrant throughout bright days and dark.

**Anti-Vaccination Tactics. The "Borax Trick."**

IF any law be found oppressive, it is quite within the rights of any citizen who feels it to be so to try by all legitimate means to obtain its repeal. Legitimate means are the avowing of his grievance; the statement of the reasons why he objects to the law in question; the publication of all facts and accurate statistics bearing on the question. If these efforts fail to convince his fellow-citizens that he is justified in his complaint, he had better cease from his futile labours, accept the inevitable—in schoolboy phrase, "grin and bear it." He is not justified, when honourable means have failed, in trying dishonourable ones—evading the law he objects to or trying to discredit it by lying statements. It is much to be wished that anti-vaccinators would keep in mind these simple canons of morality. There are honourable men among them, who go to prison with dreary regularity rather than let their children undergo an operation they consider harmful. These men may be mistaken, but they are honest, and we can respect them. But the dishonourable fathers, and still larger army of dishonourable mothers, who evade and prevaricate, and swindle doctors and magistrates, are quite another sort of person. By giving out that their children have either been vaccinated or are not susceptible to the action of the vaccine virus, they keep, among neighbours who in all probability believe vaccination to be a useful preventive, possible sources for the incubation and spread of small-pox. It can be easily done, it seems. There is, we learn from an anti-vaccination leaflet, a method casually alluded to as the "borax trick," by which the lymph will be prevented from acting, and a certificate of non-susceptibility to vaccination easily obtained from an unsuspecting doctor. This is one of the methods recommended by an anti-vaccinator, who, we are ashamed to say, holds the diploma of an honourable college. If he chooses to set himself against the general consensus of medical opinion on the subject he is free to do so; but it is wholly unjustifiable that he should teach his "borax trick" to ignorant mothers, who only feel that they do not want their plump, healthy babies to be made feverish and fretful for a week through being vaccinated. Such medical men, unscrupulous and selfish, seeking only to advertise themselves, are the most dangerous anti-vaccinators, though it is to be feared that the others—the amiable ladies who rush nto print on the strength of having clever husbands—do some mischief too. Alas! we are a nation of snobs; and it is difficult to convince the average mind that though a gentleman may have attained a certain distinction in politics or philanthropy, his wife does not, *ipso facto*, become an authority on questions of science. Still, we trust in the honesty and common sense of the nation at large to judge the vaccination question honestly, in spite of fair enthusiasts and unscrupulous charlatans. There is another phase of the anti-vaccination crusade, to which a correspondent calls attention. Certain anti-vaccinators, he declares, have formed a league, and have undertaken to write one letter each every quarter for publication in some newspaper. Let the editors be on their guard therefore, or they may be made the innocent victims of a system which, if successful, must prove harmful to the public interests. Fair criticism is one thing; the persistent reiteration of statements proved to be false and misleading is quite another.

